# Etiologic distribution of dizziness/vertigo in a neurological outpatient clinic according to the criteria of the international classification of vestibular disorders: a single-center study

**DOI:** 10.1007/s00415-023-12166-3

**Published:** 2024-01-17

**Authors:** Yue Xing, Lihong Si, Wanting Zhang, Yuru Wang, Kangzhi Li, Xu Yang

**Affiliations:** Department of Neurology, School of Clinical Medicine (Aerospace Center Hospital), Peking University Aerospace, No. 15, Yuquan Road, Haidian District, Beijing, 100049 China

**Keywords:** International classification of vestibular disorders, Neurological outpatient clinic, Dizziness, Vertigo, Etiology, Age, Sex

## Abstract

**Objective:**

The study aimed to determine the etiological characteristics of patients with dizziness/vertigo attending a neurological clinic according to the criteria of the International Classification of Vestibular Disorders (ICVD), hoping to provide a valuable reference for clinicians to diagnose and treat dizziness/vertigo.

**Method:**

A total of 638 consecutive patients with a chief complaint of dizziness/vertigo who attended the vertigo clinic of our neurology department from January 2019 to January 2020 were included. Clinical data of patients, including baseline data, medical history, neurological, neuro-otological, and auxiliary examination results were collected. The etiologic distribution of dizziness/vertigo was determined by analyzing the diagnoses of patients.

**Results:**

Of the 638 patients with dizziness/vertigo, 38.8% were males, 61.2% were females, with a male: female ratio of 1:1.58 and a mean age of 52.9 ± 16.9 years. Benign paroxysmal positional vertigo (BPPV) was the most common cause of dizziness/vertigo in both female (38.9%) and male patients (25.5%). Subgroup analysis based on sex showed that vestibular migraine (VM) and probable autoimmune inner ear disease (p-AIED) were more prevalent in female patients (10.7% and 3.8%, respectively), while vascular vertigo/dizziness was more common in male patients (10.1%). Subgroup analysis based on age showed that the most common diseases were VM in patients aged 0–30 years (27.4%), BPPV in patients aged 31–60 years (27.1%) and 61–100 years (46.0%). Episodic vestibular syndrome (EVS) was the most commonly observed, accounting for up to 60.6% (389/638) of all patients, and the most common diagnoses were BPPV (55.3%, 215/389), VM (15.2%, 59/389), primary unilateral peripheral vestibular dysfunction (p-UPVD) of unknown etiology (11.8%, 46/389), p-AIED (4.4%, 17/389), and vascular vertigo/dizziness (2.8%, 11/389) in these patients. Chronic vestibular syndrome (CVS) was found in 14.0% (90/638) of the patients, and the most common diagnoses were persistent postural-perceptual dizziness (PPPD, 35.6%, 32/90), psychogenic dizziness (18.9%, 17/90), p-UPVD of unknown etiology (15.6%, 14/90), vascular vertigo/dizziness (15.6%, 14/90), and bilateral vestibulopathy (7.8%, 7/90). Acute vestibular syndrome (AVS) was observed in 8.4% (54/638) of the patients, and the most common diagnoses were p-UPVD of unknown etiology (31.5%, 17/54), vestibular neuritis (24.1%, 13/54), probable labyrinthine apoplexy (16.7%, 9/54), stroke (13.0%, 7/54), and psychogenic dizziness (11.1%, 6/54). 16.4% (105/638) of the patients were found to have other disorders, including 15.2% (16/105) of patients with internal diseases, and 84.8% (89/105) of patients with unknown causes. In terms of localization diagnosis, 56.1%, 17.0%, 10.0%, and 16.4% of the patients were diagnosed with peripheral vestibular disorder, central vestibular disorder, psychiatric and functional vestibular disorders, and other disorders, respectively.

**Conclusion:**

(1) Dizziness/vertigo was more common in females, which was frequently caused by damage to the vestibular system. Non-vestibular or unknown etiologies were also seen in some patients; (2) VM was more prevalent in women than in men, vascular vertigo/dizziness was more commonly observed in men; (3) EVS was more common in patients with dizziness/vertigo. The most common causes of dizziness/vertigo were peripheral vestibular disorders in patients with AVS and EVS, PPPD and psychogenic dizziness in patients with CVS. The most common causes were BPPV and p-UPVD of unknown etiology in patients with a peripheral vestibular disorder, VM and vascular vertigo/dizziness in patients with central vestibular disorder, PPPD and psychogenic dizziness in patients with psychiatric and functional vestibular disorders.

## Introduction

Dizziness/vertigo is one of the most common chief complaints in neurology clinics. A previous study has shown that 3–10% of the population experienced vertigo, 17%-30% experienced dizziness, and their incidence increases with age [1]. Clinical manifestations of dizziness/vertigo are diverse, patients often present with subjective and nonspecific complaints. Additionally, dizziness/vertigo crosses multiple disciplines such as neurology, otorhinolaryngology, internal medicine, and psychiatry, the spectrum of the disease is quite complex. At present, the classification method based on the anatomical site involved and the nature of the disease is mainly used for the etiological diagnosis of dizziness/vertigo. However, for some patients with vestibular dizziness/vertigo, including peripheral vestibular disorders, central vestibular disorders, psychiatric and functional vestibular disorders, the localization and possible etiological diagnosis are difficult [2–4], this poses a challenge for clinicians to diagnose and treat the disease, resulting in a high rate of misdiagnosis and underdiagnosis [5–7].

In the chapter section of the 11th edition of the International Classification of Diseases (ICD-11)- “Diseases of inner ear”, the common causes of dizziness/vertigo are described in detail, the etiological framework is primarily based on disorders definitively affecting the vestibular labyrinth of the inner ear, structures connecting the labyrinth to the brain stem, cerebellum, subcortical structures responsible for the processing of spatial stimuli, and the vestibular cortex, these disorders can be classified into acute (AVS), episodic (EVS), and chronic vestibular syndrome (CVS). The ICD-11 states that AVS refers to a class of clinical syndromes characterized by a rapid onset, persistent vertigo, dizziness, or unsteadiness (lasting longer than 24 h, often several days and weeks), which is accompanied by nausea and vomiting, spontaneous nystagmus, and severe gait instability. The etiologies of AVS are complex, including vestibular neuritis (VN), labyrinthitis, traumatic vestibular disorders, demyelinating disorders involving the vestibule, and acute vascular vestibular dizziness. EVS is a class of clinical syndromes characterized by transient vertigo, dizziness, and unsteadiness lasting seconds to hours, occasionally days. It is usually accompanied by temporary, short-lived vestibular system dysfunction (e.g., nausea, nystagmus, and sudden falls), as well as other signs and symptoms suggestive of cochlear or central nervous system dysfunction. EVS usually connotes multiple, recurrent events caused by an episodic disorder (triggered or spontaneous), and may also initially present after the first event [8, 9]. Clinically, EVS includes benign positional paroxysmal vertigo (BPPV) [10, 11], Ménière’s disease (MD) [12, 13], vestibular migraine (VM) [14, 15], and lesions of the central or peripheral vestibular system caused by a transient ischemic attack. CVS is a group of clinical syndromes characterized by chronic dizziness, vertigo, or instability lasting from several months to several years. It is often accompanied by persistent vestibular system dysfunction (visual oscillations, nystagmus, gait instability), which includes some symptoms and signs suggestive of cochlear or central nervous system dysfunction [2, 16]. Symptoms in CVS have a progressively deteriorating course, which reflects a stable, but incomplete recovery after AVS, or represent persistent symptoms between EVS attacks. CVS includes uncompensated unilateral vestibulopathy, bilateral vestibulopathy [17, 18], cerebellar degeneration, posterior cranial fossa tumors, and psychiatric or behavioral disorders with vestibular symptoms as prominent manifestations (such as persistent postural-perceptual dizziness, PPPD) [19].

However, during the process of the diagnosis and treatment of vertigo, even if a diagnostic paradigm based on the determination of the lesion lateralization, localization, and nature of symptoms is adopted with the use of the existing vestibular evaluation techniques, it is still difficult to clarify whether the vestibular system is involved in a considerable number of patients. Moreover, the results from large-sample studies on the etiology of dizziness/vertigo vary widely [1, 20, 21], this may be related to the differences in disease severity among patients included in different studies, the level of diagnosis among hospitals of different levels, and the sample size. Given this background, this study aimed to determine the etiologic distribution of dizziness/vertigo in patients visiting the vertigo outpatient clinic of the neurology department of our hospital to validate and enrich the spectrum of dizziness/vertigo-related vestibular disorders based on the diagnostic framework as defined in the ICD-11. We hope to provide clinicians with a reference on the diagnosis and treatment of dizziness/vertigo.

## Materials and methods

### Participants

A total of 638 patients with dizziness/vertigo who visited the vertigo clinic of the neurology department of Peking University Aerospace School of Clinical Medicine (Aerospace Center Hospital) from January 2019 to January 2020 were included. The inclusion criteria included: the presence of spatial disorientation or balance disorders, including vertigo, dizziness, vestibular-visual symptoms and postural unsteadiness [2]. The exclusion criteria included: inability to cooperate with neurological, neuro-ophthalmological, neuro-otological examinations, and auxiliary tests (such as comprehensive imaging tests and vestibular function assessments).

The study was approved by the Ethics Committee of Peking University Aerospace School of Clinical Medicine (Aerospace Center Hospital). All participants included in the study provided written informed consent.

### Data collection

A detailed medical history was collected from all patients. To accurately restore the exact scene at the onset of the symptoms, the core symptoms and the background of symptom occurrence were recorded. The details about the symptoms including precipitating factors of dizziness/vertigo, symptom duration, accompanying symptoms, and the frequency of dizziness/vertigo attacks were recorded.

### Diagnosis

All patients underwent detailed physical, neurological, neuro-ophthalmological, and neuro-otological examinations, such as cranial nerve examination, Romberg’s test, Fukuda test, gaze, saccade, smooth pursuit, and optokinetic nystagmus tests, static and dynamic positional tests, bithermal caloric test, video head impulse test, vestibular-evoked myogenic potentials, and video electronystagmography. They also received auxiliary examinations, including imaging tests (such as MRI of the head or internal auditory canal, magnetic resonance angiography and CT of the head, CT Angiography of the Head and Neck, and CT perfusion), electrocochleography, immune-related laboratory test, and neuropsychological assessment using Hamilton Rating Scale for Anxiety and Depression.

Diagnoses were made by a chief senior physician according to the criteria of the International Classification of Vestibular Disorders (ICVD) when available. For vestibular disorders without established diagnostic criteria from the ICVD and non-vestibular disorders, diagnoses were made according to patients’ medical history [including onset patterns, four main vestibular symptoms identified by ICVD (dizziness, vertigo, vestibulovisual symptoms, and postural symptoms), symptom duration, triggering factors, accompanying symptoms], neurological, neuro-otological, and auxiliary examination results.

#### Diagnosis of primary unilateral peripheral vestibular dysfunction (p-UPVD) of unknown etiology

For patients with dizziness/vertigo presenting with either AVS, EVS or CVS, the diagnosis of p-UPVD of unknown etiology was made when the caloric test or the video head impulse test showed impaired vestibulo-ocular reflex function on one side, and the etiology was not identified.

#### Diagnosis of VN

We adopted more stringent criteria for the diagnosis of VN (including acute unilateral vestibulopathy and acute unilateral vestibulopathy in evolution): (1) acute or subacute onset of sustained spinning or non-spinning vertigo of moderate to severe intensity; (2) spontaneous peripheral vestibular nystagmus; (3) unambiguous evidence of reduced VOR function on the side opposite the direction of the fast phase of the spontaneous nystagmus; (4) no evidence of acute central neurological, audiological or otological symptoms or signs; (5) a history of prodromal viral infections such as shingles and pain in the ear; (6) not better accounted for by another disease or disorder [22–24].

#### Diagnosis of BPPV

BPPV is characterized by short episodes of vertigo typically lasting < 1 min. Obvious symptoms, including dizziness, lightheadedness, and unsteadiness, can be observed after changes in head position. The results from positional tests are positive. There are no hearing loss or ear disorders. BPPV can be primary or secondary to inner ear disorders and head trauma. BPPV is usually self-limited, that will resolve spontaneously without specific treatment [25].

#### Diagnosis of VM

The diagnostic criteria for definite VM include: at least five episodes of vestibular symptoms of moderate or severe intensity, lasting between 5 min and 72 h; a current or previous history of migraine with or without aura according to the International Classification of Headache Disorders (ICHD-3); headache with at least two of the following characteristics: one sided location, pulsating quality, moderate or severe pain intensity, aggravation by routine physical activity; photophobia and phonophobia; visual aura; not better accounted for by another vestibular disease or a diagnosis of the ICHD-3. The diagnostic criteria for probable VM include: at least 5 episodes with vestibular symptoms of moderate to severe intensity, lasting between 5 min and 72 h; a history of migraine or migraine features during the episode is fulfilled; not better accounted for by another vestibular diagnosis or ICHD-3 diagnosis [15].

#### Diagnosis of MD

The diagnostic criteria for definite MD include: two or more spontaneous episodes of vertigo with each lasting 20 min to 12 h; low- to medium-frequency sensorineural hearing loss during the episodes; fluctuation of auditory symptoms (hearing loss, tinnitus, feeling of fullness) in the affected ear; cannot be better explained by another vestibular disorder. Probable MD must meet the following criteria: two or more spontaneous episodes of vertigo with each lasting 20 min to 12 h; fluctuating aural symptoms (hearing, tinnitus and/or fullness) in the affected ear; cannot be better explained by another vestibular disorder [12].

#### Diagnosis of vestibular paroxysmia

The diagnosis of vestibular paroxysmia requires: at least 5 attacks of spontaneous spinning or non-spinning vertigo, lasting less than 1 min; occurs spontaneously, with or without the occurrence of postural disorders, gait disturbance, unilateral tinnitus, hearing loss on one side during the attacks; signs of neurovascular cross-over compression demonstrated on brain MRI; the response to a treatment with carbamazepine; not better accounted for by another vestibular diagnosis [26].

#### Diagnosis of PPPD

PPPD is diagnosed according to the following criteria: non-spinning vertigo, or dizziness, and unsteadiness are present simultaneously throughout the entire day for at least 3 months; symptoms occur spontaneously due to momentary postural changes or sudden movements, which are exacerbated by exposure to moving or complex visual stimuli, as well as active or passive movement. Symptoms are most severe when in an upright position, and a prone position often causes the symptoms to subside or alleviate. The most common precipitating factors of PPPD are AVS, VM, and panic attacks. The symptoms including dizziness or unsteadiness can continue for at least 3 months after these precipitating events resolved, which can cause severe anxiety or functional impairment [19].

#### Diagnosis of vascular vertigo/dizziness

The diagnosis of vascular vertigo/dizziness due to cerebrovascular disease, such as vertebrobasilar ischemia, vertebrobasilar territory transient ischaemic attacks, subclavian steal syndrome, carotid sinus syndrome, vertebral artery compression syndrome, was made by using transcranial Doppler, magnetic resonance angiography, computed tomography angiography, and digital subtraction angiography [27].

#### Diagnosis of other disorders

The diagnosis of other disorders was made based on the medical history of patients, and findings from physical, neurological, neuro-otological examinations, MRI of the head or internal auditory canal, immunologic tests and neuropsychological assessment.

### Statistical analyses

SPSS 20.0 statistical software was used for statistical analysis. Continuous data were expressed as mean ± standard deviation (SD). Normally distributed data were analyzed using the independent sample *t*-test. Categorical data was expressed as percentages, chi-square (*χ*^2^) test was used for comparison between groups, and Yates continuity correction or Fisher’s exact test was performed if necessary. All statistical significance tests were two-sided. Differences with *p* < 0.05 were considered statistically significant.

## Results

### Demographic characteristics

**Fig. 1:**
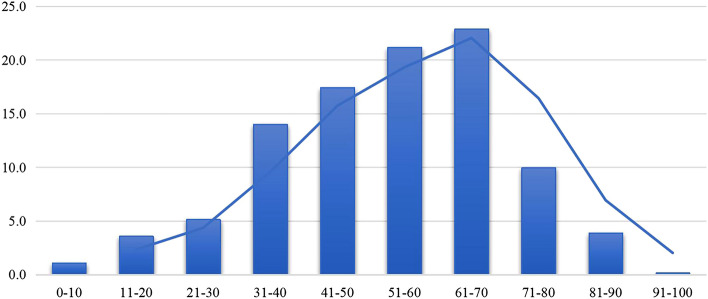
Age distribution of patients with dizziness/vertigo

**Fig. 2:**
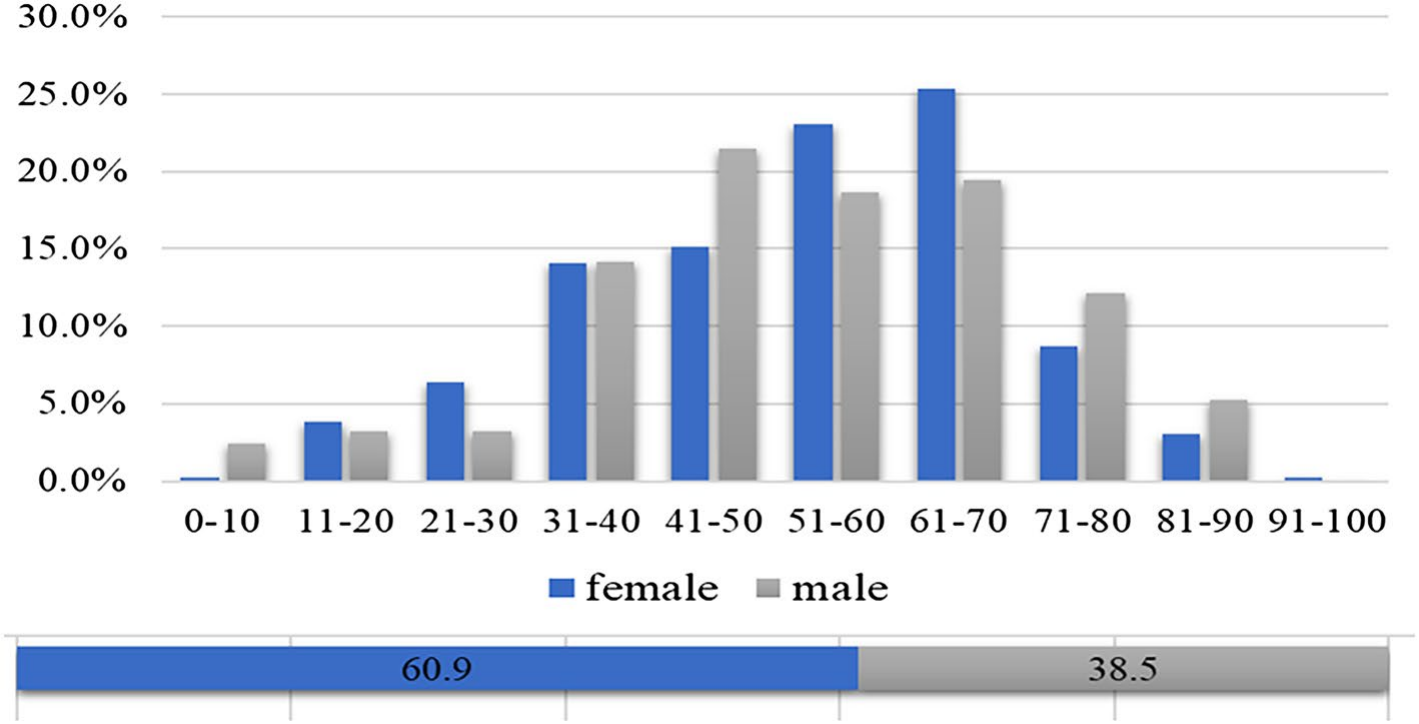
Sex distribution of patients with dizziness/vertigo in different age groups

Of the 638 patients included in the study, 38.8% (247/638) were males, and 61.3% (391/638) were females. The male: female ratio was 1:1.58. The mean age of patients was 52.9 ± 16.9 years. The highest prevalence of dizziness/vertigo was seen in patients in the age range of 41–70 years. And the mean age of male and female patients were 53.4 ± 17.6 and 52.7 ± 16.5 years, respectively. There was no statistically significant difference in age distribution between male and female patients (Figs. 1, 2).

### Etiologic distribution of dizziness/vertigo

Of the 638 patients, 33.7% (215/638) had BPPV, 13.9% (89/638) had unknown causes, 12.1% (77/638) had p-UPVD of unknown etiology, 9.2% (59/638) had VM, 6.4% (41/638) had vascular vertigo/dizziness, 4.9% (31/638) had psychogenic dizziness, 4.9% (31/638) had PPPD, 2.8% (18/638) had other neurological disorders, 2.7% (17/638) had probable autoimmune inner ear disease (p-AIED), 2.5% (16/638) had internal diseases, 2.0% (13/638) had VN, 1.6% ( 10/638) had MD, 1.1% (7/638) had other peripheral vestibular disorders, 1.1% (7/638) had bilateral vestibulopathy, and 0.8% (5/638) had vestibular paroxysmia.

### Subgroup analysis of etiologic distribution according to sex

#### Possible etiologies in female patients

Of the 391 female patients, 1.5% (6/391) had VN, 8.2% (32/391) had p-UPVD of unknown etiology, 38.9% (152/391) had BPPV, 3.8% (15/391) had p-AIED, 0.8% (3/391) had other peripheral vestibular disorders, 1.8% (7/391) had MD, 0.3% (1/391) had vestibular paroxysmia, 1.8% (7/391) had bilateral vestibulopathy, 10.7% (42/391) had VM, 4.3% (17/391) had vascular vertigo/dizziness, 1.8% (7/391) had other neurological disorders, 4.3% (17/391) had psychogenic dizziness, 4.6% (18/391) had PPPD, 2.0% (8/391) had internal diseases, and 14.6% (57/391) had unknown causes.

#### Possible etiologies in male patients

**Fig. 3:**
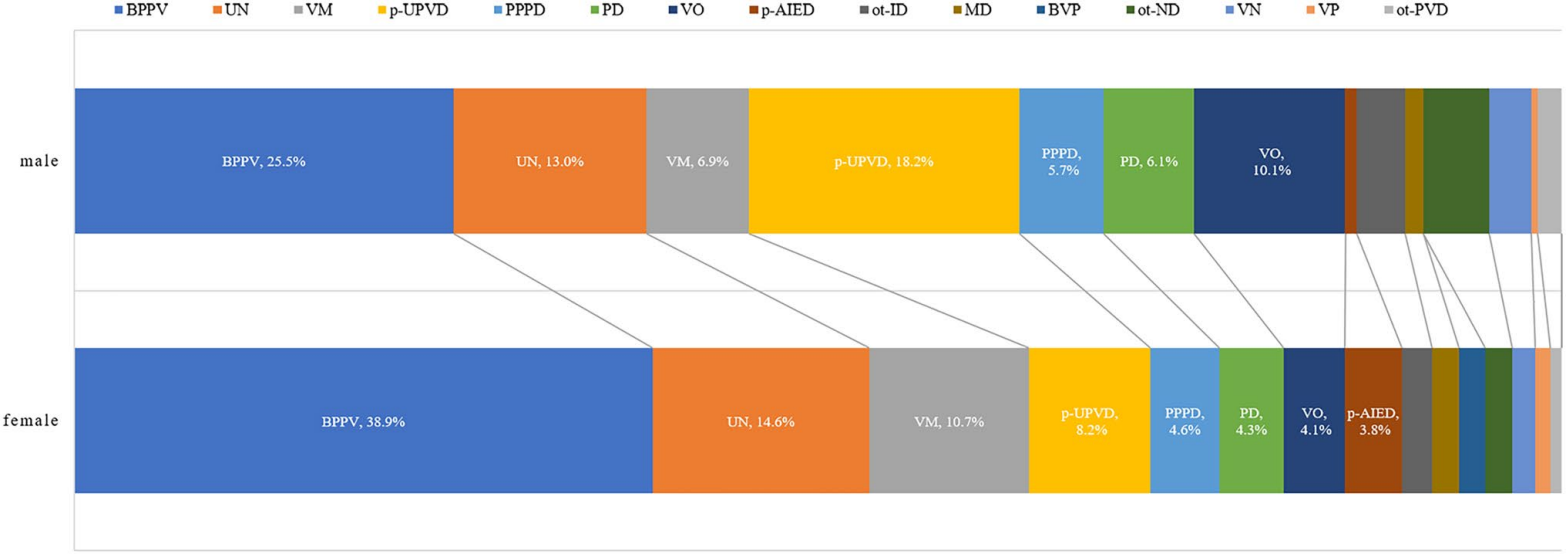
The etiologic distribution of dizziness/vertigo according to the sex of patients. *BPPV* benign positional paroxysmal vertigo; *VM* vestibular migraine; *p-UPVD* primary unilateral peripheral vestibular dysfunction of unknown etiology; *PPPD* persistent postural-perceptual dizziness; *VO* vascular vertigo and dizziness; *PD* psychogenic dizziness; *p-AIED* probable autoimmune inner ear disease; *ot-ID* other internal diseases; *MD* Ménière's disease; *BVP* bilateral vestibulopathy; *ot-ND* other neurological disorders; *VN* vestibular neuritis; *ot-PVD* other peripheral vestibular disorders; *VP* vestibular paroxysmia; *UN* unknown causes

Of the 247 male patients, 2.8% (7/247) had VN, 18.2% (45/247) had p-UPVD of unknown etiology, 25.5% (63/247) had BPPV, 0.8% (2/247) had p-AIED, 1.2% (3/247) had other peripheral vestibular disorders, 1.2% (3/247) had MD, 1.6% (4/247) had vestibular paroxysmia, 6.9% (17/247) had VM, 6.9% (17/247) had vascular vertigo/ dizziness, 4.5% (11/247) had other neurological disorders, 6.1% (15/247) had psychogenic dizziness, 5.7% (14/247) had PPPD, 3.2% (8/247) had internal diseases, and 13.0% (32/247) had unknown causes (Fig. 3).

### Subgroup analysis of etiologic distribution according to age

#### Possible etiologies in patients aged 0–30 years

9.7% (62/638) of the patients were aged 0–30 years. Among these patients, 3.2% (2/62) had VN, 9.7% (6/62) had p-UPVD of unknown etiology, 22.6% (14/62) had BPPV, 1.6% (1/62) had other peripheral vestibular disorders, 1.6% (1/62) had MD, 27.4% (17/62) had VM, 11.3% (7/62) had other neurological diseases, 4.8% (3/62) had psychogenic dizziness, 9.7% (6/62) had PPPD, and 8.1% (5/62) had unknown causes.

#### Possible etiologies in patients aged 31–60 years

53.1% (339/638) of the patients were aged 31–60 years. Of these patients, 2.4% (8/339) had VN, 12.7% (43/339) had p-UPVD of unknown etiology, 27.1% (92/339) had BPPV, 2.7% (9/339) had p-AIED, 1.2% (4/339) had other peripheral vestibular disorders, 2.7% (9/339) had MD, 0.9% (3/339) had vestibular paroxysmia, 0.6% (2/339) had bilateral vestibulopathy, 9.7% (33/339) had VM, 5.0% (17/339) had vascular vertigo/dizziness, 2.1% (7/339) had other neurological disorders, 5.6% (19/339) had psychogenic dizziness, 6.8% (23/339) had PPPD, 2.4% (8/339) had internal diseases, and 19.5% (66/339) had unknown causes.

#### Possible etiologies in patients aged 61–100 years

**Fig. 4:**
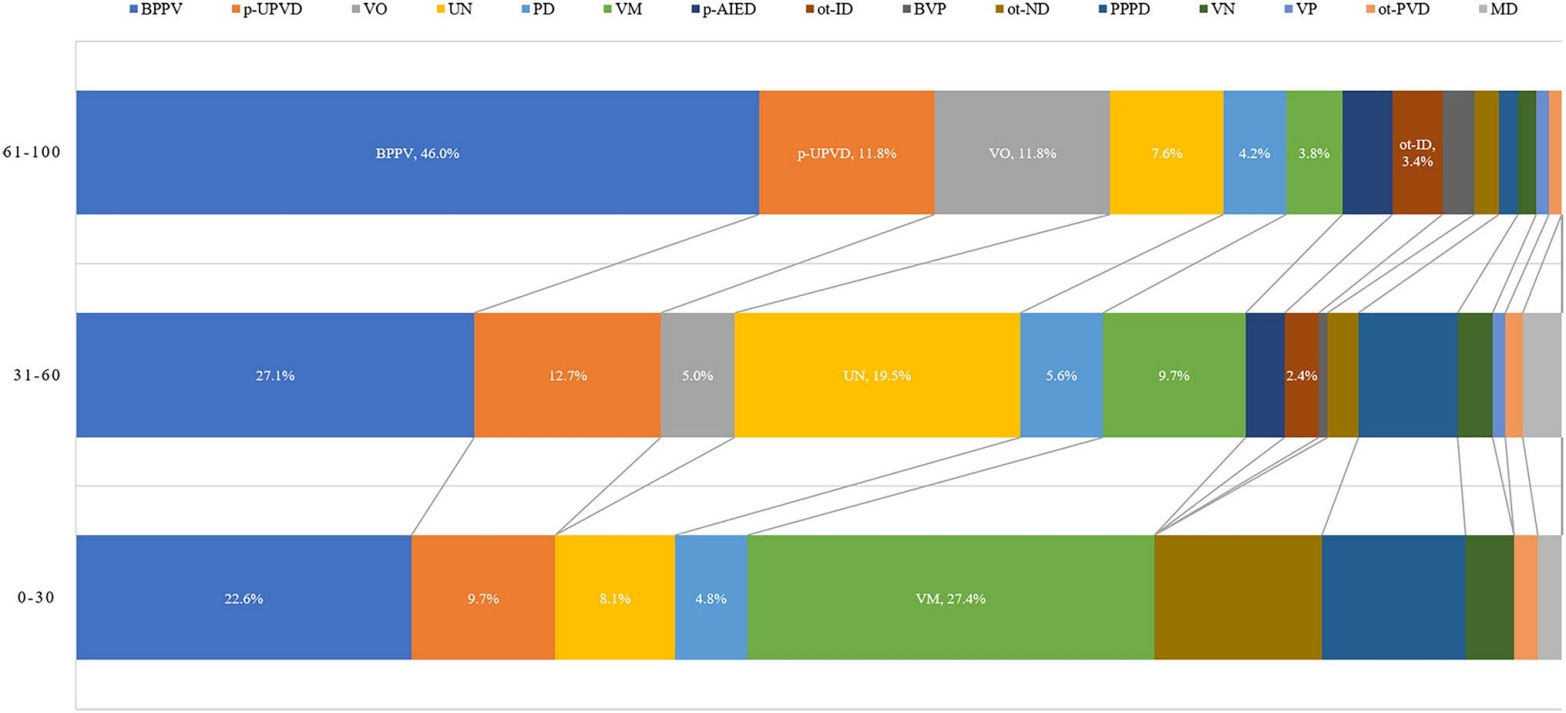
The etiologic distribution of dizziness/vertigo according to age of patients. *BPPV* benign positional paroxysmal vertigo; *VO* vascular dizziness/vertigo; *p-UPVD* primary unilateral peripheral vestibular dysfunction of unknown etiology; *PD* psychogenic dizziness; *VM* vestibular migraine; *p-AIED* probable autoimmune inner ear disease; *ot-ID* other internal diseases; *BVP* bilateral vestibulopathy; *ot-ND* other neurological disorders; *PPPD* persistent postural-perceptual dizziness; *ot-PVD* other peripheral vestibular disorders; *VP* vestibular paroxysmia; *MD* Ménière's disease; *UN* unknown causes

37.1% (237/638) of the patients were aged 61–100 years. Of these patients, 1.3% (3/237) had VN, 11.8% (28/237) had p-UPVD of unknown etiology, 46.0% (109/237) had BPPV, 3.4% (8/237) had p-AIED, 0.8% (2/237) had other peripheral vestibular disorders, 0.8% (2/ 237) had vestibular paroxysmia, 2.1% (5/237) had bilateral vestibulopathy, 3.8% (9/237) had VM, 11.8% (28/237) had vascular vertigo/dizziness, 1.7% (4/237) had other neurological disorders, 4.2% (10/237) had psychogenic dizziness, 1.3% (3/237) had PPPD, 3.4% (8/237) had internal diseases, 7.6% (18/237) had unknown causes (Fig. 4).

### Types of vestibular syndromes

Patients with AVS accounted for 8.5% (54/638) of all the patients. Of these patients, 24.1% (13/54) had VN, 31.5% (17/54) had p-UPVD of unknown etiology, 29.6% (16/54) had vascular vertigo/dizziness, 3.7% (2/54) had other neurological disorders, and 11.1% (6/54) had psychogenic dizziness.

Patients with EVS accounted for 61.0% (389/638) of all the patients. Among these patients, 11.8% (46/389) had p-UPVD of unknown etiology, 55.3% (215/389) had BPPV, 4.4% (17/389) had p-AIED, 1.8% (7/389) had other peripheral vestibular disorders, 2.6% (10/389) had MD, 1.3% (5/389) had vestibular paroxysmia, 15.2% (59/389) had VM, 2.8% (11/389) had vascular vertigo/dizziness, 2.6% (10/389) had other neurological disorders, 2.3% (9/389) had psychogenic dizziness.

Patients with CVS accounted for 14.1% (90/638) of all the patients. Of these patients***,*** 15.6% (14/90) had p-UPVD of unknown etiology, 7.8% (7/90) had bilateral vestibulopathy, 15.6% (14/90) had vascular vertigo/dizziness, 6.7% (6/90) had other neurological disorders, 18.9% (17/90) had psychogenic dizziness, and 35.6% (32/90) had PPPD.

**Fig. 5:**
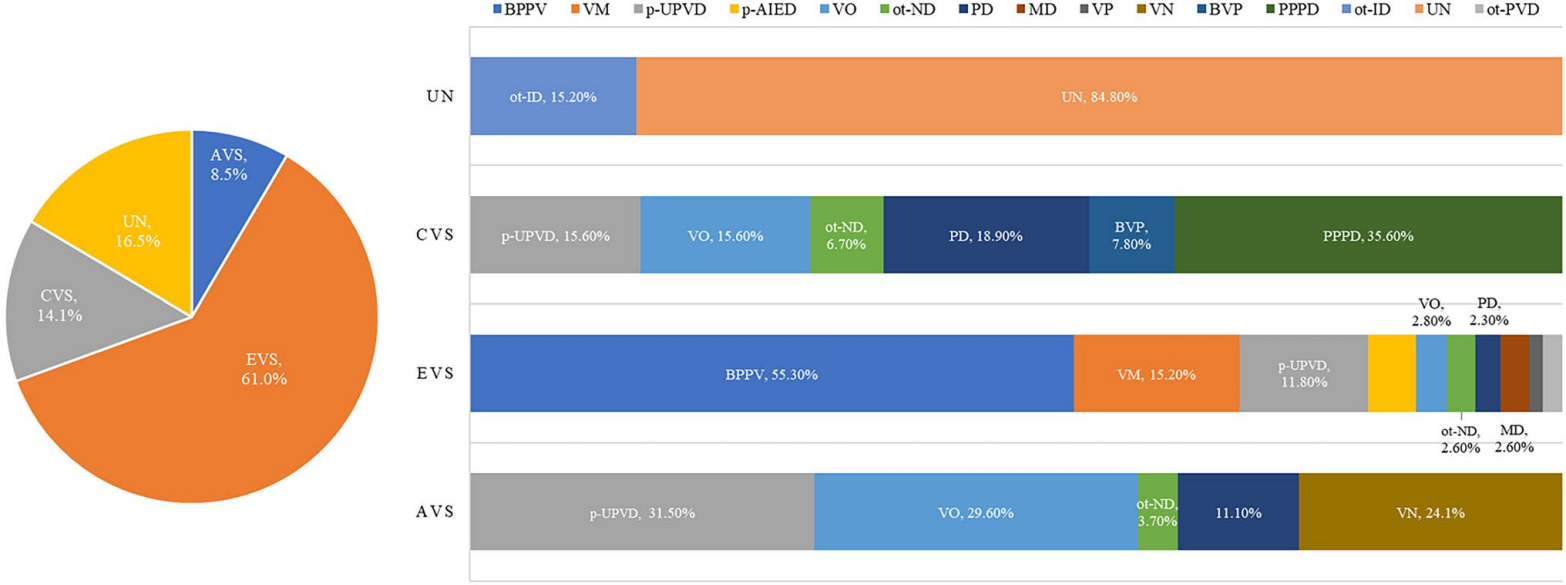
Types of vestibular syndromes and etiologic diagnosis in patients with dizziness/vertigo. *AVS* acute vestibular syndrome; *EVS* episodic vestibular syndrome; *CVS* chronic vestibular syndrome; *ot-ID* other internal diseases; *p-UPVD* primary unilateral peripheral vestibular dysfunction of unknown etiology; *VO* vascular dizziness/vertigo; *ot-ND* other neurological disorders; *PD* psychogenic dizziness; *BVP* bilateral vestibulopathy; *PPPD* persistent postural-perceptual dizziness; *BPPV* benign positional paroxysmal vertigo; *VM* vestibular migraine; *p-AIED* probable autoimmune inner ear disease; *ot-PVD* other peripheral vestibular disorders; *MD* Ménière's disease; *VP* vestibular paroxysmal; *UN* unknown causes

16.5% (105/638) of the patients had other disorders. 15.2% (16/105) of these patients had internal diseases, 84.8% (89/105) had unknown causes (Fig. 5).

### Possible localization diagnosis

56.4% (360/638) of the patients were diagnosed with peripheral vestibular disorders. 3.6% (13/360) of these patients had VN, 2.5% (9/360) had probable labyrinthine apoplexy, 21.4% (77/360) had p-UPVD of unknown etiology, 59.7% (215/360) had BPPV, 4.7% (17/360) had p-AIED, 1.9% (7/360) had other peripheral vestibular disorders, 2.8% (10/360) had MD, 1.4% (5/360) had vestibular paroxysmia, 1.9% (7/360) had bilateral vestibulopathy.

17.1% (109/638) of the patients were diagnosed with central vestibular disorders. Of these patients, 54.1% (59/109) had VM, 29.4% (25/109) had vascular vertigo/dizziness, and 16.5% (18/109) had other neurological disorders.

10.0% (64/638) of the patients were diagnosed with psychiatric and functional vestibular disorders, including 50.0% (32/64) of patients with psychogenic dizziness, and 50.0% (32/64) of patients with PPPD.

**Fig. 6:**
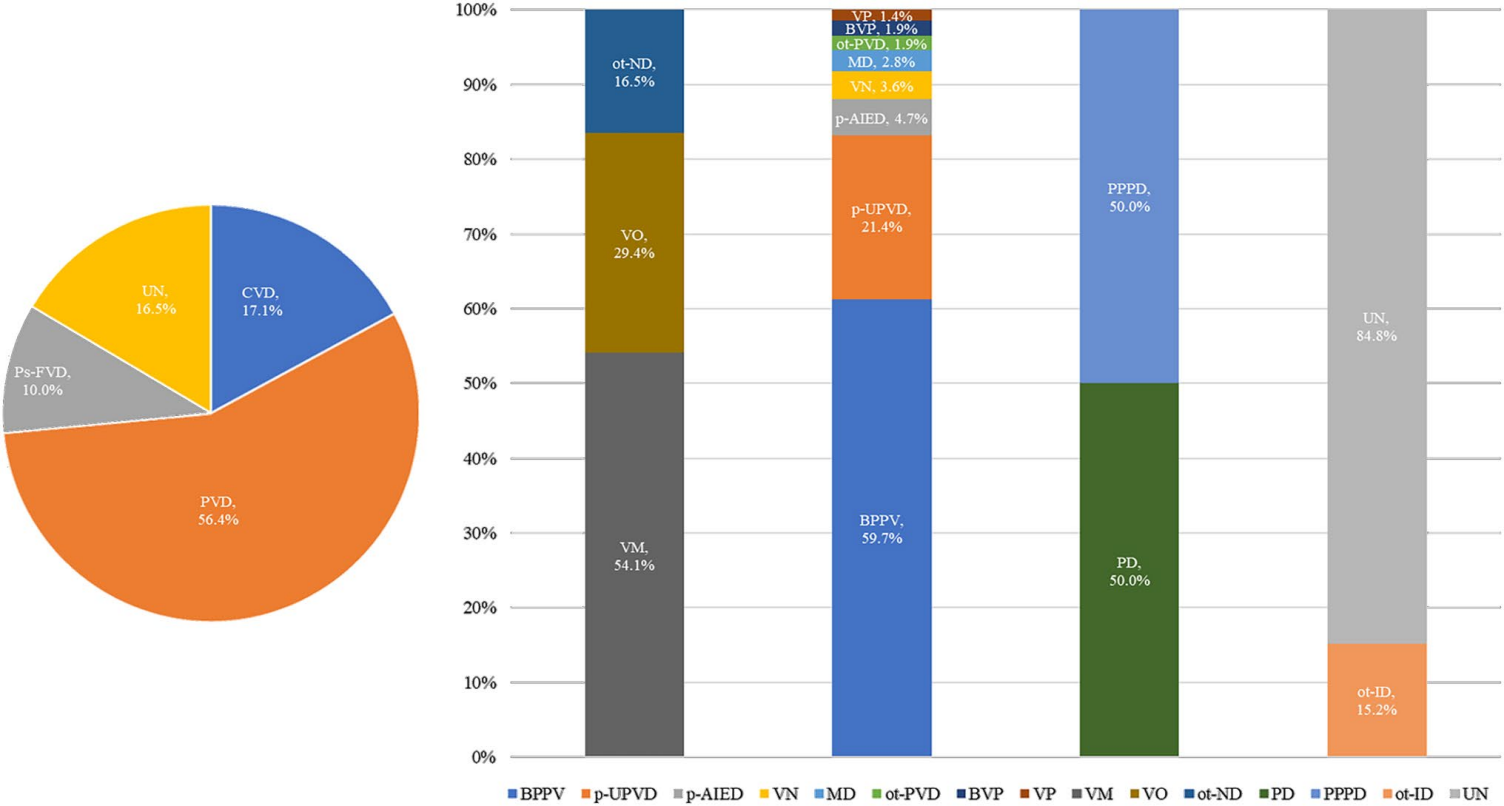
Localization and etiologic diagnosis in patients with dizziness/vertigo. *PVD* peripheral vestibular disorders; *CVD* central vestibular disorders; *PS-FVD* psychiatric and functional vestibular disorders; *OT* other; *ot-ND* other neurological disorders; *VM* vestibular migraine; *VO* vascular dizziness/vertigo; *VP* Vestibular paroxysmia; *ot-PVD* other peripheral vestibular disorders; *MD* Ménière's disease; *p-AIED* probable autoimmune inner ear disease; *BVP* bilateral vestibulopathy; *VN* vestibular neuritis; *p-UPVD* primary unilateral peripheral vestibular dysfunction of unknown etiology; *BPPV* benign positional paroxysmal vertigo; *PPPD* persistent postural-perceptual dizziness; *PD* psychogenic dizziness; *ot-ID* other internal diseases; *UN* unknown causes

16.5% (105/638) of the patients were diagnosed as having other disorders. 15.2% (16/105) of these patients had internal diseases, and the causes were unknown in 84.8% (89/105) of the patients (Fig. 6).

## Discussion

Dizziness/vertigo is one of the most common symptoms observed in all age groups, its prevalence increases with age. In this study, we determined the etiologic distribution of dizziness/vertigo according to age and sex by analyzing the diagnoses of patients with dizziness/vertigo in a neurological clinic. Diagnoses were made by determining lesion lateralization, localization, and nature of symptoms based on the ICVD criteria, as well as patients’ medical history, findings from the physical, neurological, neuro-otological and auxiliary examination. We hope to provide a reference for clinicians to diagnose and treat dizziness/vertigo.

The results of the current study showed that dizziness/vertigo can be seen in patients of all ages, with the highest prevalence being observed in the age group of 40–70 years. This finding is consistent with previous studies [28–30]. Older patients tend to have atherosclerotic risk factors such as hypertension, diabetes mellitus, and hyperlipidemia, which affect the blood supply to the inner ear, thus leading to metabolic disorder of otoconia, and age-related hormonal changes are also associated with the apoptosis of hair cells in the inner ear [28, 31]. However, in the present study, the prevalence of dizziness/vertigo showed a decreasing trend in patients aged 70 years and older. We speculate that this may be due to the fact that patients with dizziness/vertigo aged 70 and above are more likely to seek treatment or require hospitalization directly in nearby hospitals in their community, and they often have difficulty visiting high-level specialized vertigo center that is located far from their own reach. In subgroup analysis with age stratification, BPPV was found to be the most common cause of dizziness/vertigo in patients of all ages, but the proportion of each cause differed among patients of different ages. VM was more common in patients under the age of 30 years, and vascular vertigo/dizziness was relatively frequent in patients aged 60 years and older.

In the present study, we found that dizziness/vertigo was more common in females than in males, which is consistent with previous studies [21, 32]. The results of this study showed that BPPV was the most common cause of dizziness/vertigo in both female and male patients. VM and p-AIED were more commonly observed in female patients. Previous studies revealed that women are more prone to VM, this may be due to the influence of the female endocrine system, VM has genetic predisposition displaying autosomal dominant inheritance, and reduced penetrance in males [33]. Females are more likely to have an autoimmune disease than males, and it is estimated that up to 78% of people affected by autoimmune diseases were females [34]. AIED can be either primary organ-specific damage of the inner ear, or a non-specific symptom with inner ear involvement due to systemic autoimmune disorders (such as Wegener’s granulomatosis, polyarteritis nodosa, and systemic lupus erythematosus) [35]. It is believed that estrogen and progesterone exert potent regulation effects on the immune system, and can clear virus more rapidly, whereas androgens exert suppressive effects on the immune system. Therefore, women have stronger protective immunity, loss of self-tolerance can occur more frequently, thus resulting in the development of autoimmune diseases [36]. The results of the present study showed that vascular vertigo/dizziness was more commonly seen in male patients, the possible reason may be due to that a higher proportion of males than females reported cardiovascular risk factors such as smoking and alcohol consumption [37].

In the present study, we found that EVS was more commonly observed in patients with dizziness/vertigo attending the neurological outpatient clinics, accounting for up to 60.6% of all patients, followed by CVS (14.0%), and AVS was less common in these patients. The reason may be that our center is a higher-level specialized vertigo center of a tertiary hospital, the majority of patients with AVS seek treatment from local hospitals or hospital emergency departments, and patients who were referred to the tertiary hospital mostly have CVS or EVS. The results of this study showed that VN, p-UPVD of unknown etiology, and vascular vertigo/dizziness were the most common causes of dizziness/vertigo in patients with AVS. In patients with EVS, BPPV, VM, and p-UPVD of unknown etiology were more commonly seen, p-AIED, MD, vestibular paroxysmia, and other rare types of peripheral vestibular diseases were also observed. p-UPVD of unknown etiology was more common in patients with CVS, we speculate that CVS mainly includes uncompensated unilateral and bilateral vestibulopathy, cerebellar degeneration, and psychiatric disorders with vestibular symptoms as prominent manifestation, these findings are consistent with previous studies [16, 38]. From the above results, we can see that the diagnosis of vestibular syndromes can help narrow the list of differential diagnoses and make more accurate diagnosis.

Through the determination of the lesion localization, we found that peripheral vestibular disorders was more common in patients with dizziness/vertigo, accounting for 56.1% of all patients, followed by central vestibular disorders (17.0%), other disorders (16.4%), and psychiatric and functional vestibular disorders (10.0%). For patients with peripheral vestibular disorders, BPPV was the most prevalent, followed by p-UPVD of unknown etiology, which may be associated with vascular, immune, and inflammatory-mediated mechanisms. For patients with central vestibular disorders, VM was the most prevalent, followed by vascular vertigo/dizziness. PPPD and psychogenic dizziness were the most commonly observed in patients with psychiatric and functional vestibular disorders. These findings are similar to the results of a large-sample study on etiologies of patients with dizziness/vertigo and balance disorders form Germany showing that peripheral vestibular disorders, central vestibular disorders, psychiatric and functional vestibular disorders, other disorders, and unknown causes accounted for 45.2%, 25.1%, 17.6%, 8.3%, and 3.7% of all patients, respectively, the most common causes of dizziness/vertigo were BPPV (15.5%), MD (10%), unilateral peripheral vestibulopathy (8.9%), bilateral peripheral vestibulopathy (6.7%), and vestibular paroxysmia (3.4%) in patients with peripheral vestibular disorders, which were central dizziness/vertigo (13.3%) and VM (11.8%) in patients with central vestibular disorders. A similar study also found that peripheral vestibular disorders were more common in patients with dizziness/vertigo, BPPV was the most common diagnosis, MD, unilateral vestibulopathy, psychiatric and functional vestibular disorders (psychogenic dizziness or/PPPD) were the second most common diagnoses, and the central causes of dizziness/vertigo mainly included vascular disorders (brainstem and cerebellar infarcts), VM, and multiple sclerosis [20]. These findings suggest that the etiologies of dizziness/vertigo are extremely complex, including peripheral, central vestibular disorders, and psychiatric disorders. However, in contrast to the findings of multicenter studies from Germany [32] and Korea [21], our results showed that the proportions of VN, MD, other rare types of peripheral vestibular disorders, bilateral vestibulopathy, and vestibular paroxysmia were relatively low. We speculate that the possible reason for this discrepancy is that patients included in these studies were from different outpatient settings, patients presenting to the neurology and otorhinolaryngology outpatient clinics have a high proportion of central and peripheral dizziness/vertigo, respectively, and non-vestibular dizziness/vertigo caused by systemic diseases is more commonly seen in patients presenting to the emergency department. Additionally, the proportion of patients with VN (2%) was only a little bit higher than the proportion of patients with MD (1.6%). The reason is twofold. Firstly, most patients with VN are treated in primary medical institutions, with reduced number of patients referred to our high-level specialized vertigo center. Secondly, more stringent diagnostic criteria was used for making a diagnosis of VN.

It is noteworthy that the causes of dizziness/vertigo were unknown in many patients, accounting for about 13.9% of all patients. The reason may be due to the fact that most patients visiting our center had complex conditions with relatively long disease duration, and some patients were referred multiple times, often making diagnosis difficult. A combination of medical history, findings from physical examination, multidimensional vestibular tests (such as electronystagmography, caloric tests, video head impulse test, cervical vestibular evoked myogenic potentials), non-vestibular evaluation techniques (such as post-contrast delayed 3D-FLAIR imaging), and even functional magnetic resonance imaging techniques can be used to determine the lesion localization. If necessary, dynamic follow-up can be performed on patients to further confirm the diagnosis. Furthermore, in this study, we found that dizziness/vertigo caused by internal diseases of the system accounted for about 3.4% of all the patients. Previous studies reported that dizziness/vertigo is most commonly caused by vestibular dysfunction, which can also be caused by other systemic diseases [39, 40]. Therefore, accurate identification of non-vestibular vertigo, assessment of disease severity specialists, and timely referral of patients by vestibular medicine are required.

### Limitations

The study has some limitations. First, this was a single-center study conducted at a vertigo outpatient clinic of a tertiary hospital. Although our center is a high-level specialized vertigo center, patients visiting our center were from all over the country, the selection and referral bias may be inevitable. Second, owing to relative small sample size, BPPV was not classified according to the involved canals and the underlying pathophysiological mechanism (canalithiasis or cupulolithiasis), the proportion of patients with two or more diagnoses was not analyzed, and disorders with a very low proportion (such as orthostatic hypotension or postural orthostatic tachycardia syndrome), were categorized into other disorders. Muti-center and large-scale studies with in-depth stratified analyses are therefore needed to further confirm our findings.

## Conclusions

Our findings suggest that dizziness/vertigo was more common in females, which was frequently caused by damage to the vestibular system, and non-vestibular or unknown etiologies were also seen in some patients.VM was more prevalent in women than in men, whereas vascular vertigo/ dizziness was more commonly observed in men. EVS was more common in patients with dizziness/vertigo. The most common causes of dizziness/vertigo were peripheral vestibular disorders in patients with AVS and EVS, PPPD and psychogenic dizziness in patients with CVS. The most common diagnoses were BPPV and p-UPVD of unknown etiology in patients with peripheral vestibular disorders, VM and vascular vertigo/dizziness in patients with central vestibular disorders, PPPD and psychogenic dizziness in patients with psychiatric and functional vestibular disorders.

## Data Availability

The datasets generated and/or analyzed during the current study are not publicly available due to privacy and ethical restrictions but are available from the corresponding author on reasonable request.
