# Correction to: Etiologic distribution of dizziness/vertigo in a neurological outpatient clinic according to the criteria of the international classification of vestibular disorders: a single‑center study

**DOI:** 10.1007/s00415-024-12495-x

**Published:** 2024-07-23

**Authors:** Yue Xing, Lihong Si, Wanting Zhang, Yuru Wang, Kangzhi Li, Xu Yang

**Affiliations:** Department of Neurology, Aerospace Center Hospital, Peking University Aerospace School of Clinical Medicine, No. 15, Yuquan Road, Haidian District, Beijing, 100049 China

**Correction to:** **Journal of Neurology (2024) 271:2446–2457** 10.1007/s00415-023-12166-3

In the original version of this article, affiliation details for authors Yue Xing, Lihong Si, Wanting Zhang, Yuru Wang, Kangzhi Li and Xu Yang were incorrectly given as

Department of Neurology, School of Clinical Medicine (Aerospace Center Hospital), Peking University Aerospace, No. 15, Yuquan Road, Haidian District, Beijing 100049, China

but should have been

Department of Neurology, Aerospace Center Hospital, Peking University Aerospace School of Clinical Medicine, No. 15, Yuquan Road, Haidian District, Beijing 100049, China

